# Manual and Mechanical Induced Peri-Resuscitation Injuries—Post-Mortem and Clinical Findings †

**DOI:** 10.3390/ijerph191610434

**Published:** 2022-08-22

**Authors:** Daniel Gödde, Florian Bruckschen, Christian Burisch, Veronika Weichert, Kevin J. Nation, Serge C. Thal, Stephan Marsch, Timur Sellmann

**Affiliations:** 1 Department of Pathology and Molecularpathology, Helios University Hospital Wuppertal, University Witten/Herdecke, 58455 Witten, Germany; 2 Department of Anaesthesiology and Intensive Care Medicine, Evangelisches Krankenhaus BETHESDA zu Duisburg, 47053 Duisburg, Germany; 3 State of North Rhine-Westphalia/Regional Government, 44145 Düsseldorf, Germany; 4 Department of Trauma Surgery, Berufsgenossenschaftliche Unfallklinik Duisburg, 47249 Duisburg, Germany; 5 NZRN, New Zealand Resuscitation Council, Wellington 6011, New Zealand; 6 Department of Anaesthesiology I, University Witten/Herdecke, 58455 Witten, Germany; 7 Department of Anesthesiology, HELIOS University Hospital, 42283 Wuppertal, Germany; 8 Department of Intensive Care, University Hospital, Petersgraben 4, 4031 Basel, Switzerland

**Keywords:** manual/device-assisted cardiopulmonary resuscitation, injuries/adverse effects, etiology, quality improvement, adult/child, retrospective studies

## Abstract

(1) Background: Injuries related to resuscitation are not usually systematically recorded and documented. By evaluating this data, conclusions could be drawn about the quality of the resuscitation, with the aim of improving patient care and safety. (2) Methods: We are planning to conduct a multicentric, retrospective 3-phased study consisting of (1) a worldwide literature review (scoping review), (2) an analysis of anatomical pathological findings from local institutions in North Rhine-Westphalia, Germany to assess the transferability of the review data to the German healthcare system, and (3) depending on the results, possibly establishing potential prospective indicators for resuscitation-related injuries as part of quality assurance measures. (3) Conclusions: From the comparison of literature and local data, the picture of resuscitation-related injuries will be focused on and quality indicators will be derived.

## 1. Introduction

Manual and/or mechanical cardiopulmonary resuscitation (CPR) with chest compressions and ventilations maintains minimal organ perfusion in the event of cardiac arrest (CA). CPR averts the immediate death of the patient until circulatory function is reestablished by return of spontaneous circulation (ROSC). The current standard CPR technique was initially described in the early 1960s [1,2,3] and has become the gold standard treatment for all patients in CA.

CPR is a frequent event, which if successful can be associated with considerable follow-up costs. Worldwide over 135 million cardiovascular deaths occur each year with an increasing prevalence in coronary heart disease cases [4]. The incidence of out-of-hospital cardiac arrest (OHCA) ranges from 20 to 140 per 100,000 people [5]. In 2019, the German Resuscitation Register [6] data from 88 rescue services showed 15,274 patients with OHCA and ROSC and 3741 patients with in-hospital cardiac arrest (IHCA) and ROSC from 118 participating hospitals. These numbers represent approximately 18% of treated CA patients [7], meaning that approximately 60,000 CPR procedures occur per year.

Cardiopulmonary resuscitation may initially achieve ROSC, but the overall survival rates are low and range from 2% to 11% [5]. In order to improve the incidence of ROSC, national and international standardized guidelines have been established [8,9,10]. However, even if delivered according to these guidelines, CPR is often inefficient [11], providing only 10–30% of normal blood flow to the heart and 30–40% of normal blood flow to the brain [12,13,14,15]. With high quality chest compressions (CC) blood flow can be reestablished by up to 90% [11,16,17,18,19]. Therefore, high quality CPR is key to improve patient survival [20] even though it may result in injuries [21].

Despite the potentially lifesaving properties of CC, the key feature is repeated blunt force trauma to the chest, which can lead to extensive traumatic skeletal and non-skeletal injuries [22]. Cardiopulmonary resuscitation involves vigorous, rhythmic chest compressions performed manually or with the aid of a device. The reported incidence of CPR-associated injuries ranges from 21% to more than 65% [21].

The duration of CPR has been associated with the number of rib fractures and the occurrence of sternal fractures [23]. The duration of CPR in OHCA management has been identified as an independent risk factor for chest injuries, possibly due to the difficulty of maintaining the adequate quality of CPR [24]. Other aspects of CPR, such as hand position during CC [24,25,26], CC rate [27,28], CC depth [29,30,31,32,33], or chest wall recoil [34,35] are still under debate.

Injuries related to CPR can affect the skeletal system, as well as soft tissue and organs. Commonly, fractures occur of the ribs and sternum. Less often, CPR causes fractures of the larynx or injury to the stomach, spleen, heart, or liver [36,37]. Gastric injuries are less frequent [38,39,40] but are a potentially lethal complication after initial successful resuscitation [40]. Resuscitation-associated major liver injury, such as rupture or laceration, and hemorrhage or hematoma are also rare and indicate compromised hemostasis with no significant influence on the overall outcome [41]. Heart injury, rupture of the heart, pericardial tamponade, and aortic dissection have been reported [37,42] as well as lung contusion [42], lung herniation [43], and upper airway and pulmonary injuries [44].

In patients with ventricular fibrillation or pulseless ventricular tachycardia, the rapid use of automatic external defibrillator has a positive impact on survival [45,46,47] but may cause skin injuries [48,49] or cardiac and renal damages due to rhabdomyolysis [37].

Risk factors for injuries include female gender [50,51] and older age [42,51]. Skeletal, organ and relevant soft tissue injuries occur very rarely after pediatric CPR [52,53,54]. Even when performed by nonmedical or untrained individuals, CPR did not increase the likelihood of injury in the pediatric population [55]. In adults, sternal fractures occur in at least one-fifth of cases and rib fractures and/or sternal fractures in at least one-third of patients during conventional CPR [56], sometimes causing a flail chest [57,58].

Usually, CPR is performed manually, although external mechanical assist devices may also be available [59,60,61,62]. Mechanical CPR devices may provide a useful adjunct to standard treatment in situations where high-quality manual CC cannot be provided [20]. However, the use of devices during CPR can also lead to injuries. In an animal model, the LUCAS™ device shows superior resuscitation outcomes and less thoracic injuries, compared to the Corpuls CPR [63]. However, in forensic autopsy studies, the use of LUCAS™ compared with manual CPR was associated in part with significantly higher rates of sternal fractures, rib fractures, and severe soft tissue injuries, including several case reports of potentially life-threatening injuries [64,65,66,67]. In comparison, the patterns of trauma in autopsy records between AutoPulse^®^ CPR and manual CPR showed different characteristic patterns of injuries. Manual CPR showed a characteristic pattern with higher frequencies of anterior rib fractures, sternal fractures, and midline chest abrasions along the sternum. AutoPulse^®^ CPR-associated injuries included a high frequency of posterior rib fractures and skin abrasions located along the anterolateral chest and shoulder and a few cases of visceral injuries [68]. The use of CardioPump^®^ showed an increased frequency of thoracic injuries following CPR [69]. The incidence of skeletal injuries increased with patient age along with the use of CPR devices [70] (Table 1).

In addition to clinical findings and radiological diagnostics, autopsies also play an important role in the identification of CPR-associated injuries [50,69,71,72,73]. Autopsies show the highest diagnostic sensitivity. Both frequent skeletal injuries and the rarer injuries to internal organs are reliably detected by autopsy [51,74,75,76]. Postmortem diagnostics can also be used to answer questions regarding injury patterns or the impacts of CPR related injury on survival [55,68].

The aim of our study is to further contribute to the understanding of CPR-associated injuries. To do this, data from published international medical literature will be compared with local clinical and post-mortem data. We expect to fill knowledge gaps regarding a perceived discrepancy between the expected and actual incidence of resuscitation-associated injury patterns. We will report their extent, as well as their influence on outcome. Our results will inform the development of possible indicators for improving CPR quality.

CPR technique continues to be developed. To date, original papers and systematic reviews focus on individual aspects of CPR. In addition, there is significant heterogeneity in study design and research methodology [56,77]. Although current guidelines address CPR quality, strategies to systematically monitor CPR quality are rare [16,78]. Here, the influence of CPR feedback and CPR training could contribute to improve CPR quality [79,80,81]. In other fields of medicine, the use of a systematic continuous quality improvement approach has been shown to optimize outcomes [82,83,84].

## 2. Materials and Methods

### 2.1. Study Design

This is a retrospective study undertaken following the Declaration of Helsinki principles and after approval by the local Ethics Committee of the Witten/Herdecke University. The study is divided into three sections: a scoping review, a local national retrospective multicenter evaluation, and potentially, an evolution of quality indicators based on the results of our previous findings.

#### 2.1.1. Literature Review

The review protocol was developed using the statement and checklist of Preferred Reporting Items for Systematic reviews and Meta-Analyses extension for Scoping Reviews (PRISMA-ScR) (30178033). The search strategy was developed by all authors (TS, DG, CB, KN, FB, VW, ST, SM), with detailed search terms subsequently generated. To identify potentially relevant documents, the following bibliographic databases are searched: MEDLINE, CINAHL, EMBASE, ClinicalTrials, and Cochrane Library. The review will include all kinds of published academic peer-reviewed research articles, review articles, and case reports in English and German except unpublished “gray” literature. The search strategy for MEDLINE is structured as follows: (“Heart arrest” [All Fields] OR “cardiac arrest” [All Fields]) AND (“cardiopulmonary resuscitation” [All Fields] OR “CPR” [All Fields] OR “chest compression” [All Fields]) AND (“manual” [All Fields] OR “hands” [All Fields] OR “hands-only” [All Fields]) AND (“Mechanical” [All Fields] OR “LUCAS” [All Fields] OR “AutoPulse” [All Fields] OR “Load distributing band” [All Fields]) AND (“injury” [All Fields] OR “injuries” [All Fields] OR “trauma” [All Fields] OR “violation” [All Fields] OR “hurt” [All Fields] OR “lesion” [All Fields] OR “harm” [All Fields] OR “findings” [All Fields]) AND (“post mortem” [All Fields] OR “dead” [All Fields] OR “deceased” [All Fields] OR “autopsy” [All Fields]). The search results will be exported in Rayyan (www.rayyan.ai, accessed on 15 May 2022) and duplicates will be removed. All titles and abstracts are split up and screened in teams of two reviewers independently. Disagreements will be resolved via discussion, with reference to the a priori eligibility criteria until consensus is achieved. Residual data are collected and documented if they meet the inclusion criteria (Table 2).

#### 2.1.2. Local Retrospective Multicenter Evaluation

The results from our scoping review will be compared to the results of autopsies undertaken in the last 10 years in participating universities and private pathology laboratories, as well as forensic medicine departments in the state of North Rhine-Westphalia. These will be analyzed to determine the occurrence of mechanic traumatic resuscitation-related injuries. The data from this regional review will be analyzed to determine recommendations for quality improvement and their application more broadly across Germany.

#### 2.1.3. Evolution of Quality Indicators

Derived key variables from the collected review data will be used to suggest parameters to evaluate resuscitation outcomes as part of quality management systems. These key variables could be used to determine criteria for data collection in the German Resuscitation Register.

### 2.2. Data Analysis

Statistical data analysis will be performed using the open-source software “PSPP” [85]. Data will be presented as mean ± standard deviation (SD) or percentages. Wherever it is appropriate, *t*-test will be used for the comparison of means between groups and Pearson’s chi-squared test will be used to check the independence between categorical variables. *p* values less than 0.05 are considered statistically significant.

## 3. Conclusions

To improve the quality of CPR, all sub-areas and possible complications of this complex procedure need to be examined in detail. Although CPR is primarily about restoring blood flow, and thus the perfusion of the organs, it is ultimately the outcome that determines the success. It is, therefore, of interest to not only optimize the process and performance of CPR but also analyze possible risk factors and complications. The knowledge of possible injuries, frequency of occurrence, severity, and risk factors can lead to prevention strategies for both health professional and lay rescuers. Following the use of CPR devices, which may be accompanied by more frequent and severe injuries, consideration should be given to a patient clinical examination. Thus, complications for the patient, some of which can be serious, can be reduced and follow-up costs minimized.

By standardizing the process of CPR together with a continuous training of potential professional and lay rescuers, the quality of CPR can be proven [79,80,81]. To date, certain quality assurance parameters have already been identified [86] and established [16]. Complications, such as CPR-associated injuries, have been previously discussed, but number, frequency, and severity have not yet been described in a consistent manner. Furthermore, to date, no quality indicators have been derived from findings.

It is the aim of this study to investigate and develop a map of possible CPR-associated injuries. We propose that the knowledge of the possible types of CPR-associated injuries, frequency of occurrence, and degree of impact on CPR and outcome can be used to develop strategies of avoiding the occurrence of such injuries. In addition, a classification of the CPR-associated injuries into minor and major groups will not only simplify therapeutic decisions after CPR but also guide mitigation strategies. In the identification and weighting of CPR-associated injuries, postmortem findings play an important role in addition to clinical data [50,71,72,73,87]. The combining of clinical and postmortem data can further inform the likely severity of injuries. We hypothesize that our comparison of local data with findings from the literature will help fill in gaps in knowledge.

This study has several limitations. First, both the scoping review and multicenter evaluation are based on retrospective data. Furthermore, the scoping review will include only papers in English and German and no analysis of the quality of the included papers will be performed. In addition, we acknowledge limitations in our search strategy and that the local multicenter evaluation covers a limited period.

## Figures and Tables

**Table 1:** CPR-related injuries and risk factors associated with CPR devices.

CPR Device	CPR-Related Injuries	Risk Factors
LUCAS^TM^	Rib and sternal fracture; haemato- and pneumothorax; pulmonary contusion; lung bleeding; pulmonary edema; mediastinal, retrosternal, and pericostal bleeding; hematoperitoneum; aortic laceration; right coronary artery rupture; cardiac petechiae; epicardial hemorrhage; myocardial rupture; hemopericardium; laceration of esophageal, tracheal, and gastric mucosae; pancreatic bleeding; perirenal bleeding; liver and spleen laceration; chest cutaneous lesions; breast implant rupture	Positioning/handling the device, CPR duration, age, female gender
AutoPulse^®^	Rib and vertebral fractures; skin abrasions; liver, mesenteric, and splenic laceration; hemoperitoneum	
CardioPump^®^	Vertebral, sternal, and rib fractures; skin abrasions; liver laceration	

**Table 2:** Inclusion and exclusion criteria for the scoping review.

**Inclusion criteria**	human (male/female, any gender, any age)
	clinical or non-clinical resuscitation
	manual or mechanical resuscitation
	any type of injuries
	any anatomical region of injuries
	any number of injuries
	any underlying diseases
	any procedure to determine injuries
	any patient outcome
**Exclusion criteria**	animal model
	resuscitation without injuries
	injuries resulting from inadequate perfusion/ventilation
